# Beyond skin white spots: Vitiligo and associated comorbidities

**DOI:** 10.3389/fmed.2023.1072837

**Published:** 2023-02-23

**Authors:** Zhonghui Hu, Tao Wang

**Affiliations:** Department of Dermatology, State Key Laboratory of Complex Severe and Rare Diseases, Peking Union Medical College Hospital, Chinese Academy of Medical Sciences and Peking Union Medical College, National Clinical Research Center for Dermatologic and Immunologic Diseases, Beijing, China

**Keywords:** Vitiligo, comorbidities, etiology, pathogenesis, melanocytes

## Abstract

Vitiligo is a common depigmentation disorder of an unknown origin characterized by the selective loss of melanocytes, resulting in typical white macules and patches. However, vitiligo is now recognized as more than just a skin disease, what a dermatologist observes as a white spot of skin is just the “tip of the iceberg” of the condition. We attempt to clarify the classification of comorbidities associated with vitiligo from various reviews and reports, and describe their possible pathogenesis. In conclusion, the literature provides evidence of an association between vitiligo and ocular and auditory abnormalities, autoimmune disorders, other dermatological diseases, metabolic syndrome and related disorders, and psychological diseases. These associations highlight the importance of a multidisciplinary approach in managing vitiligo patients.

## 1. Introduction

**Figure 1:**
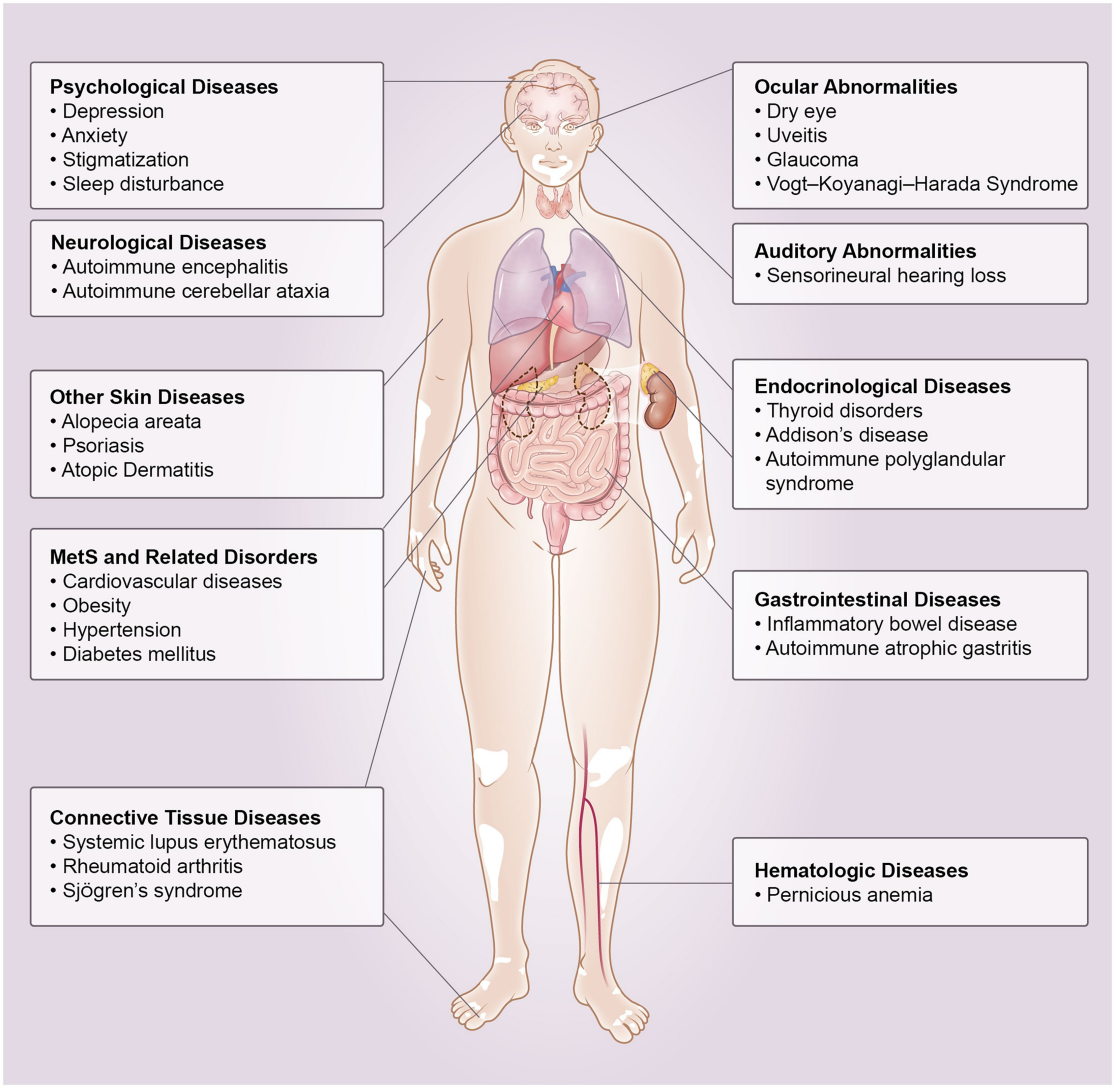
Comorbidities associated with vitiligo. MetS: metabolic syndrome.

**Figure 2:**
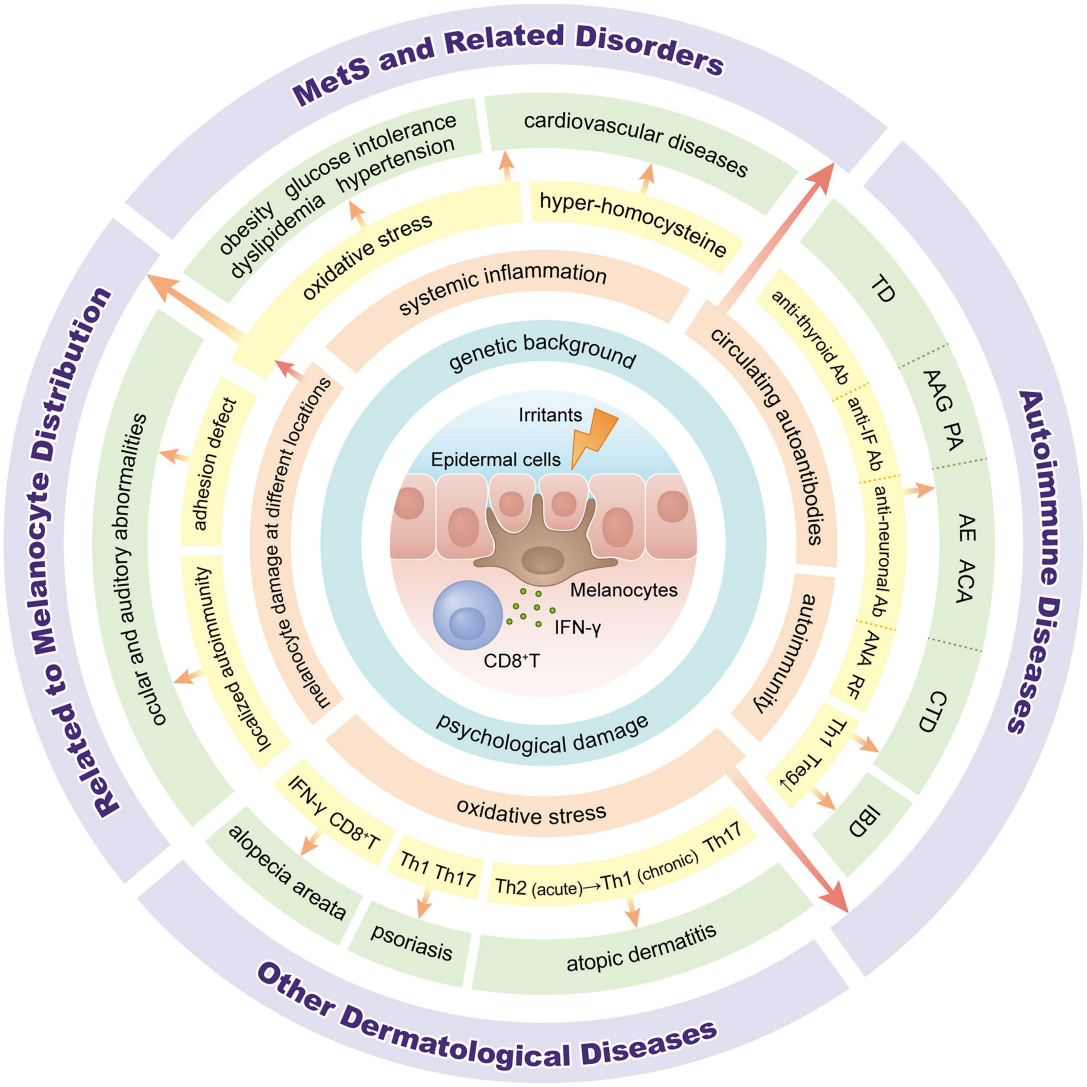
Overview of pathogenesis between vitiligo and associated comorbidities. IFN: interferon; Ab: antibody; IF: intrinsic factor; ANA: anti-nuclear antibody; RF: rheumatoid factor; Th: T-helper; Tregs: regulatory T cells; TD: thyroid disease; AAG: autoimmune atrophic gastritis; PA: pernicious anemia; AE: autoimmune encephalitis; ACA: autoimmune cerebellar ataxia; CTD: connective tissue diseases; IBD: inflammatory bowel disease; MetS: metabolic syndrome.

Vitiligo is a chronic inflammatory autoimmune condition that results in skin depigmentation due to the loss of melanocytes (1, 2). Globally, the prevalence ranges from 0.5 to 2.0% and varies geographically (3). The pathogenesis of vitiligo is not completely understood. Many theories, such as genetic background, autoimmune responses, oxidative stress, melanocyte adhesion, and neuronal involvement, have been proposed for the pathogenesis of vitiligo (2, 4). However, vitiligo is now recognized as more than just a skin disease, some studies have found that vitiligo is associated with several organ-specific or systemic disorders, including ocular or otologic diseases, autoimmune disease, metabolic syndrome (MetS) and psychological diseases (5–8). In this review, we clarify the classification of comorbidities associated with vitiligo and describe their possible pathogenesis (Figures 1, 2).

We searched the electronic database PubMed, Web of Science, Embase and Cochrane Library from inception to April 30, 2022 and analyzed the relevant literature related to vitiligo and associated comorbidities, including case–control, cross-sectional, and cohort studies, as well as systematic reviews and meta-analyses. A combination of medical subject headings (MeSH) and free terms were used in the search. The MeSH terms included “vitiligo,” “eye diseases,” “ear diseases,” “autoimmune diseases,” “metabolic syndrome” and “mental disorders.”

## 2. Related to melanocyte distribution

Epidermal melanocyte damage is one of the causes of vitiligo. However, in addition to the skin, melanin and melanocytes are also present in the eyes, cochlea, leptomeninges, heart, and even inhospitable environments, such as adipose tissue (9). Therefore, some comorbidities associated with vitiligo may be related to melanocyte damage at different locations.

### 2.1. Ocular abnormalities

Melanocytes are found in both the retinal pigment epithelium (RPE) and the choroid of the eye, with the RPE originating from the neural ectoderm and the latter from the neural crest (10). Melanocytes in the RPE are crucial for the metabolism of rod outer segments and retinoids and photoprotection of the retina, whereas melanocytes in the choroid contribute to eye pigmentation and UV protection (9, 11). Damage to these melanocytes and reduced melanogenesis may result in ocular abnormalities and even vision impairments.

Prabha et al. (12) found various ocular abnormalities, such as hypopigmented trabecular meshwork, pigment clumps, uveitis, and RPE atrophy, in patients with vitiligo, and periorbital depigmentation was associated with eye abnormalities. Another cross-sectional study by Genedy et al. (13) revealed a significantly higher prevalence of ocular abnormalities in patients with vitiligo but no significant differences in visual acuity, which may be because ocular melanocytes are not directly involved in detecting or transferring visual information. There have also been reports of vitiligo comorbidities that cause loss of vision. For example, Dertlioğlu et al. (14) found that among 49 patients with vitiligo, 9 patients (18.4%) had normal-tension glaucoma (NTG), whereas there were no signs of NTG in the control group. In the absence of treatment, NTG can cause permanent loss of vision because it is a chronic progressive neuropathy that damages the optic nerve. Moreover, Rogosić et al. (15) confirmed primary open-angle glaucoma in 24 of 42 patients (57%) with vitiligo suspect of glaucoma. Multivariate logistic regression revealed that advanced age and a long vitiligo duration were risk factors for primary open-angle glaucoma.

According to one study by Ma et al. (7), patients with vitiligo had significantly reduced tear production, shorter tear film break-up time, and more symptoms related to dry eyes, which may be attributed to localized autoimmunity, such as T-helper (Th) 17 cells and related cytokines, concomitant rheumatologic diseases and adhesion defect theory (16–19). Additionally, subfoveal choroidal thickness was significantly thinner in the vitiligo group, possibly reflecting melanocyte depletion within this structure. Several external irritants, such as trauma, toxic chemical agents, ultraviolet radiation, and infection, can trigger oxidative stress, resulting in excessive reactive oxygen species production, autoimmune reactions, and melanocyte death (4, 20). It is also evident from the Köebner phenomenon that friction and trauma play critical roles in the pathogenesis of vitiligo (21). These stress signals spread to melanocytes in other parts of the body, potentially increasing their comorbidity risk.

### 2.2. Auditory abnormalities

In the auditory system, melanocytes are distributed in the stria vascularis and spiral ligament of the cochlea, which are required for maintaining the endocochlear electrical potentials that play an essential role in normal hearing (22). Melanin has been demonstrated to reduce oxidative stress by scavenging free radicals, and oxidative stress is considered one of the most important factors underlying sensorineural hearing loss (23, 24).

Accordingly, numerous studies have examined the relationship between vitiligo and hearing impairment but with varying results (25–28). A recent systematic review indicated that patients with vitiligo had a 6.02-fold increased risk of developing sensorineural hearing loss compared with the control group, but the definition of hearing loss was heterogenous between included studies (29). Lien et al. (6) conducted a similar analysis, and only studies meeting the criteria that examined hearing loss by assessing the pure-tone thresholds (PTTs) were included. According to their research findings, patients with vitiligo had significantly elevated PTTs only at high frequencies, including 2,000, 4,000, and 8,000 Hz, not at low frequencies ranging from 250 to 1,000 Hz. Since most of the sound in our daily life are at low and intermediate frequencies, we are generally not sensitive to hearing damage in the high frequency region. As a result, most cases of hearing loss among patients with vitiligo are subclinical. Furthermore, Anbar et al. (30) conducted a study to compare cochlear function in patients with nonsegmental vitiligo (NSV) and segmental vitiligo (SV), and the results showed that bilateral cochlear dysfunction was common in both disease subtypes. Regarding the age of onset, one study revealed a tendency towards an increased severity of sensorineural hearing loss in the older group and patients with late-onset vitiligo (28). In contrast, another study found that late-onset vitiligo was not statistically associated with abnormalities of the auditory system (27).

Based on the findings of the above studies, we recommend regular evaluation of patients with vitiligo to detect ocular and auditory abnormalities and to treat these conditions in the early stage. Melanocyte destruction in patients with vitiligo can affect melanocytes that share a common embryological origin, ultimately affecting the function of the organs in which they reside. However, small sample sizes and heterogeneous definitions have led to conflicting results between these studies. Further large-scale studies are required to clarify the disease burden and the associated pathogenesis of ocular and auditory abnormalities in patients with vitiligo.

### 2.3. Vogt–Koyanagi–Harada syndrome

Vogt–Koyanagi–Harada syndrome (VKH) is an autoimmune disease that mainly affects melanocyte-containing systems, such as the eyes, ears, meninges, skin, and hair follicles (31). It is characterized by granulomatous uveitis with varying degrees of extraocular manifestations, such as headache, meningismus, hearing loss, poliosis, and vitiligo (32). According to the current theory, VKH is an exacerbated response by melanocytes and their precursor cells compared with vitiligo. The study by Egbeto et al. (33) supports the theory that T cell responses (particularly cytotoxic CD8+ T cells), type 1 cytokines, memory T cell responses, and chemokines are involved in the development of VKH and vitiligo.

Melanocytes are also located in the brain and leptomeninges, possibly with neuroendocrine and detoxification functions (9). Melanocytes are also present in the heart, where they may play a role in electrical signaling (34). However, there are few clinical studies of diseases associated with melanocyte destruction in these regions, and the correlation between these diseases and vitiligo requires further study.

## 3. Autoimmune diseases

There is an extensive correlation between vitiligo and other organ-specific or generalized autoimmune disorders. A 10-year retrospective study involving 3,280 patients showed that comorbid autoimmune conditions occur in approximately 23% of vitiligo patients, including thyroid disease (TD), rheumatoid arthritis (RA), inflammatory bowel disease (IBD), systemic lupus erythematosus (SLE), and type 1 diabetes mellitus (35). Of note, a cross-sectional study showed that patients with at least one comorbid autoimmune disease tended to have more extensive vitiligo compared with those without comorbid autoimmune disease (36).

As mentioned above, the exact pathogenesis of vitiligo remains complicated and unclear, but the most accredited hypothesis is the autoimmune theory, which may help explain how the disease manifests systemic alterations. First, vitiligo patients show circulating autoantibodies directed toward melanocyte antigens. Clinical studies also found that a significant number of patients tested had elevated anti-nuclear antibody titers, rheumatoid factor positivity, and increased anti-thyroid antibodies compared to the general population (35, 37–41), which imply potential cross-talk between vitiligo and associated diseases. Second, C-X-C motif chemokine ligand 10 (CXCL10), a marker of Th1-mediated immune responses, is elevated in peripheral liquids from vitiligo patients (42). Recent studies have shown that serum and tissue CXCL10 expression are increased in organ-specific autoimmune diseases, such as autoimmune thyroiditis, Graves’ disease, type 1 diabetes, and systemic rheumatological disorders (RA, SLE, systemic sclerosis, mixed cryoglobulinemia), underlining the importance of a common immunopathogenesis of these disorders characterized by a Th1-prevalent autoimmune response (43, 44). Third, regulatory T cells (Tregs) play a critical role in the augmentation of peripheral immune tolerance, thereby protecting the human body from autoimmune damage (45). Accumulating data suggests that patients with vitiligo have a reduced number of Tregs; decreased Treg suppression; and reduced levels of Treg-associated suppressive cytokines, such as interleukin (IL)-10 and transforming growth factor-β (46, 47). Likewise, quantitative and functional deficiencies in Tregs have been reported in a variety of autoimmune diseases, including RA, SLE, type 1 diabetes mellitus, multiple sclerosis, and myasthenia gravis, among others, which might contribute to the elevated frequency of various associated autoimmune diseases in patients with vitiligo (48). Fourth, vitiligo lesions tend to recur at the same location of previous depigmented patches after treatment cessation, suggesting a potent role of non-recirculating tissue-resident memory T (TRM) cells in the pathogenesis of vitiligo (49, 50). TRM cells respond rapidly to the secondary exposure of known antigens *in situ* and provide efficient protection. They also respond to autoantigens in other barrier tissues, including the lungs, gastrointestinal tract, and reproductive tract, as well as in non-barrier tissues, including the brain, pancreas, and joints, which may be involved in the pathogenesis of organ-specific autoimmune disorders, such as IBD, multiple sclerosis, and type 1 diabetes mellitus (51). Finally, through genetic studies, over 50 vitiligo-associated genes and loci have been discovered, and many have also been found to be associated with other autoimmune diseases (52). Thus, these genetic associations may confirm the shared underlying genetic predisposition between vitiligo and other autoimmune diseases. In summary, patients with vitiligo are more likely to suffer from autoimmune conditions than the general population.

### 3.1. Endocrinological diseases

#### 3.1.1. Thyroid disorders

Among all autoimmune diseases associated with vitiligo, TD is one of the most common and widely studied (5). Conversely, recent research has demonstrated that vitiligo is one of the most prevalent autoimmune diseases among patients with autoimmune thyroiditis (53). The British guidelines suggest that dermatologists should be aware of the increased risk of TD or autoimmune thyroid disease (ATD) in vitiligo patients, and routine examination of thyroid function and antibodies should be considered (54).

A recent meta-analysis of 37 studies with 78,714 vitiligo patients showed that in all patients with vitiligo, the prevalence of TD, ATD, thyroid peroxidase antibodies (TPOAbs), and thyroglobulin antibodies (TGAbs) was 15.7, 1.9, 16.8, and 11.4%, respectively, which was significantly higher than that in healthy controls (40). Another systematic review involving 77 studies reported by Yuan et al. (55) assessed the prevalence of six thyroid disorders in vitiligo patients, including subclinical hyperthyroidism, overt hyperthyroidism, subclinical hypothyroidism, overt hypothyroidism, Graves’ disease, and Hashimoto thyroiditis. The highest prevalence was in subclinical hypothyroidism, and the lowest was in subclinical hyperthyroidism or Graves’ disease. In addition to serum thyroid autoantibodies (TPOAbs, TGAbs, and thyroid-stimulating hormone receptor antibodies), which are closely associated with thyroid autoimmune disease, Colucci et al. (56) evaluated thyroid hormone antibodies (THAbs) against triiodothyronine and/or thyroxine and found surprisingly high levels in patients with vitiligo. Furthermore, they suggested that THAbs may act as a “bridge of vicious cycles” between melanocytic and thyroid systems. Moreover, a cohort study involving 700 patients summarized the clinical characteristic of generalized vitiligo patients with TDs (57). According to their results, the affected body surface area (BSA) was significantly higher in patients with vitiligo combined with TD. Notably, these patients also had a typical distribution pattern of lesions; the most common depigmentation areas were the hands, wrists, ankles, and elbows, which are susceptible to friction (classified as Köebner phenomenon type 2A).

An increasing number of studies focusing on the type and onset of vitiligo or gender and race of patients aim to identify the confounding factors based on subgroup analyses. Many studies have confirmed that NSV patients are more susceptible to TD than SV patients, possibly because SV is more associated with neural mechanisms (58). Some studies divided vitiligo patients into early-onset and late-onset groups and defined the former as onset before 12 years of age (59). They found that early-onset vitiligo patients exhibited a lower prevalence of TD compared with the late-onset group, potentially because the latter might be more associated with acquired autoimmunity (40, 60). Sexual dimorphism of the immune system exists between men and women (61, 62). van Geel et al. (57) reported that female patients with vitiligo more frequently have TD compared with male patients, and this phenomenon might be associated with stimulation of the autoimmune system by estrogen in women.

#### 3.1.2. Addison’s disease

A significant correlation was found between vitiligo and Addison’s disease. Alkhateeb et al. (63) reported that the prevalence of Addison’s disease was 0.38% among Caucasian vitiligo probands and 0.087% among their relatives, which is substantially higher than the prevalence in the general population. Similarly, in another study involving 113 family members of Addison’s disease patients, 3.5% were diagnosed with vitiligo, which was significantly more frequent than the proportion of controls (64).

Autoimmune polyglandular syndrome (APS) occurs when two or more endocrine glands in the same individual become hypofunctional because of autoimmune inflammation, either sequentially or simultaneously (65). It can also affect the non-endocrine system. APS-1 is a rare recessive inherited disease caused by autoimmune regulator gene mutations and characterized by chronic mucocutaneous candidiasis, chronic hypoparathyroidism, and Addison’s disease (66). A recent study analyzed autoimmune conditions associated with autoantibodies in 158 Italian APS-1 patients. At the end of follow-up, 17% had vitiligo, and melanin-producing cell autoantibodies were found in 10/18 tested patients (67). APS-2 is characterized by adrenal insufficiency (Addison’s disease), ATD, or type 1 diabetes mellitus and has also been reported to be significantly associated with vitiligo (68).

### 3.2. Gastrointestinal diseases

#### 3.2.1. IBD

IBD is an immune-mediated chronic intestinal inflammatory disease and includes Crohn’s disease (CD) and ulcerative colitis (UC). Some large-scale studies found a higher frequency of IBD among vitiligo patients, but the prevalence varies according to country and ethnicity. Hadi et al. (69) reported that 1.1% of their vitiligo patients had IBD, which is 2.13-fold higher than the incidence in the general population. Sheth et al. (35) reported a considerably higher prevalence of IBD in their 3,280 vitiligo patients in the USA. Among the 2.3% of patients with IBD, 1.4% had UC, 0.6% had CD, and 0.3% were unspecified. Furthermore, Alkhateeb et al. (63) showed that the frequency of IBD was elevated by 2-fold, with 0.67% among adult Caucasian vitiligo probands compared with a population frequency of 0.37%. Conversely, Jo et al. (70) analyzed 64,837 patients with IBD in the Korean population, and after adjusting for age and insurance type, patients with IBD had a higher risk of vitiligo than non-IBD subjects.

#### 3.2.2. Autoimmune atrophic gastritis

Autoimmune atrophic gastritis (AAG), characterized by the development of antibodies against parietal cells and intrinsic factor, leads to mucosal destruction that primarily affects the corpus and fundus of the stomach (71). Vitiligo has been described in association with AAG, but most studies were case reports (71, 72). Only one retrospective study involving 138 AAG patients showed that vitiligo was present in 2.8% of patients (73). Further studies are required to understand the associations between vitiligo and AAG.

### 3.3. Hematologic diseases

Several observational studies have found an increased prevalence of pernicious anemia (PA) in the vitiligo population. A 10-year cross-sectional retrospective study of 1,487 vitiligo patients in urban USA showed that an estimated 0.4% of patients have combined PA (69). Another study in 1,098 patients in the USA suggested a similar incidence of approximately 0.5% (36). It is worth noting that the prevalence of PA was significantly increased in a study conducted in Canada, with 1.3% of 300 vitiligo patients reporting the disease (74), which was higher than the general population prevalence of 0.15%. However, in a similar population-based study that enrolled 14,883 vitiligo patients in Taiwan, no statistically significant association of PA with vitiligo was revealed (57). This lower prevalence may be responsible for the differences in comorbidity profiles between Easterners and Westerners.

PA occurs in the later stage of AAG with severe gastric intrinsic factor deficiency and consequent vitamin B12 deficiency (75). PA is linked to but different from AAG. Anti-parietal cell antibodies (APCAs) are a serum biomarker of AAG present in most patients with AAG and 85–90% of individuals with PA (76), and APCAs are more frequent among individuals with vitiligo (77). Hence, associations of vitiligo with AAG and PA indicate a common immunopathogenic pathway.

### 3.4. Connective tissue diseases

The most commonly reported comorbid connective tissue diseases (CTD) in vitiligo patients are SLE and RA. Other associated CTDs include Sjögren’s syndrome (SS), systemic sclerosis (SSc), and dermatomyositis/polymyositis (DM/PM).

Choi et al. (78) performed a large-scale cross-sectional study and indicated that 86,210 patients with vitiligo were at an increased risk of SLE, SSc, SS, and RA. Subgroup analysis showed an increased risk of DM/PM for males and ankylosing spondylitis for female vitiligo patients. Research conducted by Gill et al. (36) in a US population found a statistically significant higher prevalence of SLE (0.3%), SS (0.2%), discoid lupus (0.2%), and linear morphea (0.2%) in vitiligo patients, and SLE was observed only in Black patients. Another similar study in vitiligo patients in the United States reported a considerably higher prevalence, with 2.2% for SLE and 2.9% for RA. Stratified analyses based on race/ethnicity suggested that these two comorbidities were more common in the African-American/Black population, which is consistent with the previous study (35). In Taiwan, a case–control study also showed a significant association of vitiligo with SLE and SS. In the age- and gender-stratified analysis, only patients with onset between 60 and 79 years of age were found to display an increased risk of SLE (79). In summary, age-, sex-, or ethnicity-specific approaches for comorbid CTDs in vitiligo patients will assist in the proper management of these disorders by clinicians.

### 3.5. Neurological diseases

Recent studies have suggested an observational link between vitiligo and neuroimmune disorders involving the peripheral and central nervous systems. According to a cross-sectional analysis of 1,098 patients with vitiligo, three had Guillain-Barré syndrome (0.2%), two had multiple sclerosis (0.2%), and two had myasthenia gravis (0.2%) (36). Considering that both the skin and the nervous system originate from the ectoderm, the underlying pathogenesis may be related to the fact that the epitopes of the neuronal cell surface are more likely to be attacked by autoantibodies induced by exposure to antigens triggered by vitiligo.

Autoimmune encephalitis is a group of disorders characterized by antibodies reacting with the extracellular epitopes of neuronal cell membranes or synaptic proteins (80). Of the three cases reported by Ren et al. (81), two suffered from anti-leucine-rich glioma-inactivated 1 encephalitis, and one had anti-IgLON5 encephalopathy. A Mayo Clinic cohort study showed that in a series of 62 patients with anti-glutamate decarboxylase 65 antibody-positive autoimmune cerebellar ataxia (ACA), 10 patients (16%) had vitiligo (82). In addition, Han et al. (83) reported a patient with ACA with anti-delta/notch-like epidermal growth factor-related receptor antibodies who suffered from vitiligo for more than 20 years. Most published studies on the association of vitiligo with neurological disease are case series or small sample studies. Therefore, further studies are warranted to investigate the cross-links between them.

Based on the above, we speculate that vitiligo may be a clue to understanding the diagnosis or autoimmune etiology of other autoimmune disorders. Patients with vitiligo are prone to disruption of autoimmune tolerance and autoimmune attack, thereby increasing their risk of concomitant autoimmune diseases. Similarly, if vitiligo lesions are observed in patients with diseases of other tissues, autoimmunity may be responsible for the pathogenesis. A diagnosis based on this information may be more accurate. Several large cross-sectional or retrospective studies have also noted that these diseases often occur alongside one another, which implies that their pathologies are inextricably linked. As a result, vigilance should be exercised when searching for possible concomitant diseases, and patients should be referred to specialists where necessary. Nevertheless, further studies are required to determine whether therapies for vitiligo can slow the progression of concomitant autoimmune disorders.

## 4. Other dermatological diseases

### 4.1. Alopecia Areata

Vitiligo and alopecia areata (AA) are common autoimmune conditions characterized by white spots on the skin (vitiligo) and bald spots on the scalp (AA) (84). A retrospective study of 1,098 patients with vitiligo showed that AA is the second most common autoimmune disease associated with vitiligo after ATD, occurring in 3.8% of patients with vitiligo (36). Notably, a relatively high AA prevalence of 5.3% was observed in a study involving 133 NSV patients in Japan (85). Conversely, a Danish nationwide register-based cohort study showed that a diagnosis of AA was significantly associated with a higher risk of vitiligo (86), and the same phenomenon has been reported in pediatric patients (87).

Oxidative stress and autoimmunity with genetic susceptibility are associated with the pathogenesis of AA and vitiligo (88). Both conditions are characterized by a prominent interferon (IFN)-γ + signature and cytotoxic CD8 + T cell attack, selectively targeting the anagen hair follicle bulbs in AA and the epidermal melanocytes of the basal layer in vitiligo (84). Additionally, Tomaszewska et al. (89) found elevated levels of the oxidative stress markers IFN-γ, IL-1β, and IL-6 in the serum of both NSV and AA patients. In clinical practice, oral Janus kinase inhibitors are a promising treatment with demonstrated effectiveness in AA and vitiligo patients (90, 91). Together, these facts indicate a similar pathogenesis of both diseases.

### 4.2. Psoriasis

Psoriasis is a relatively common disease with an estimated prevalence ranging from 0.51 to 11.43% in adults (92). A recent systematic review showed that compared with controls, psoriasis patients were 2.29-fold more likely to have vitiligo, and vitiligo patients were 3.43-fold more likely to be diagnosed with psoriasis (93). However, regarding its incidence in vitiligo patients, there is a large heterogeneity across studies. In a descriptive and cross-sectional study, 7.79% of 154 vitiligo patients simultaneously had psoriasis (94). However, in a similar study in 712 vitiligo patients, only 3% of the vitiligo group had associated psoriasis (95). In another cross-sectional study, in addition to the current comorbidities, Canu et al. (96) evaluated the history of psoriasis, and the results showed that 16.9% of 463 vitiligo patients had a past and/or current personal history of psoriasis. Remarkably, a case–control study demonstrated that inflammation or pruritus in vitiligo macules and a family history of cardiovascular disease were the most significant predictors of patients having both psoriasis and vitiligo (97).

Shared cell-mediated immune pathogeneses, including Th1 and Th17 pathways activated by IFN-γ, may play a role in the similar patterns of autoimmune inflammation in both diseases (98). Moreover, genome-wide association studies have provided extensive evidence that psoriasis and vitiligo share common genetic variants with similar effect sizes, including allelic variations in genes related to immune responses, such as *AIS1*, *PSOR7* (99), and the major histocompatibility complex (100).

### 4.3. Atopic dermatitis

Increasing evidence suggests that vitiligo is associated with atopic dermatitis (AD). A meta-analysis of 16 studies showed that patients with vitiligo had a 7.82-fold increase in AD compared with control patients without these disorders. Intriguingly, the odds of having AD were higher in those with early-onset vitiligo than in those with adult-onset vitiligo (101). Similarly, the results of a recent population-based cohort study of 173,709 patients newly diagnosed with AD showed that people with AD had a higher incidence of vitiligo compared with the general population, particularly when AD is more severe (102). In addition, Roh et al. (103) conducted a study to characterize the real-world comorbidities associated with adult AD. They also found that AD was associated with increased odds (odds ratio = 4.44) of vitiligo, and the same findings were confirmed in another identical study in a pediatric population (104). Furthermore, AD prevalence was stratified by vitiligo BSA, and the results showed that a BSA of 76% or higher was associated with an increased risk of AD (105).

There are a few possible explanations for the pathomechanism linking AD and vitiligo. Campione et al. (106) suggested that although AD is associated with a Th2-mediated immune response in its acute phase, its chronic phase is predominantly Th1-mediated with remodelling and fibrosis of the tissues. In addition, Th17 cells, which produce IL-17A and IL-17F, seem to play a role in AD by interacting with eosinophils and triggering the Th2 response, which are also involved in the development of vitiligo (107). What’s more, Silverberg et al. (105) proposed that the proinflammatory state of AD may lead to melanocyte destruction, while scratching of pruritic AD lesions may köebnerize vitiligo.

Vitiligo and other associated dermatological diseases are characterized by a complex combination of factors, including genetic predisposition and the immune response triggered by exogenous stimulation. Vitiligo can precede or co-occur with other skin comorbidities, or emerge at different stages of the development of other skin comorbidities. Despite these obvious clinical differences, skin diseases share much in common, and understanding their similarities may help us to better determine their pathogenesis and develop common therapeutic approaches to treat them in a more efficient way, such as biological agents or Janus kinase inhibitors.

## 5. MetS and related disorders

MetS is described as the clustering of obesity, hypertension, hyperglycemia, and dyslipidemia in an individual, ultimately leading to diabetes mellitus, cardiovascular diseases, or other chronic diseases (108). Several clinical or fundamental studies have investigated the association of vitiligo with MetS or its components.

Recently, Chuang et al. (8) conducted a meta-analysis and revealed a significant association between vitiligo and MetS (odds ratio = 1.648). Furthermore, in subgroup analyses based on the type and activity of vitiligo, MetS prevalence was not significantly different. Another recent meta-analysis of 30 studies found significant associations between vitiligo and MetS components, including diabetes mellitus, obesity, and hypertension. Kang et al. (109) found that patients with vitiligo had a 3.30-fold increased risk of developing diabetes mellitus and a 2.08-fold increased risk of suffering from obesity compared with controls, and the prevalence of hypertension was 19.0% in vitiligo patients. A meta-analysis conducted by Chang et al. (110) demonstrated that vitiligo was significantly associated with type 1 and type 2 diabetes mellitus. These meta-analysis findings are consistent with the results of some previous case–control studies. Tanacan et al. (111), Ataş et al. (112), and Sharma et al. (113) found that the frequency of MetS was higher in patients with vitiligo compared with that in control groups. Furthermore, Tanacan et al. (111) and Ataş et al. (112) reported a higher rate in patients with the active or severe form of the disease, whereas Sharma et al. (113) showed that MetS remained unaffected by the severity of vitiligo.

MetS and its components are important risk factors for cardiovascular disease. Carotid ultrasound measurements, including carotid intima media thickness (CIMT), are used for the assessment of subclinical atherosclerosis and can be independent predictors of cardiovascular events (114). A hospital-based, case–control study among Egyptians by Azzazi et al. (115) showed that a significantly higher proportion of vitiligo patients had hypercholesterolemia and borderline high, high, or very high levels of low-density lipoprotein. Atherosclerotic plaques and increased CIMT were detected significantly more in patients than in controls. In addition, the CIMT was significantly correlated with the Vitiligo Area Severity Index and duration of vitiligo (116). Intriguingly, another study by Karadag et al. (117) demonstrated higher levels of homocysteine in vitiligo patients than in controls. Homocysteine inhibits tyrosinase, an enzyme involved in melanin synthesis and a marker for cardiovascular diseases.

There are some similarities in the underlying pathophysiology of vitiligo, MetS, and atherosclerosis, including genetic background, pro-inflammatory signaling pathways, and increased oxidative stress. Genetically, a genome-wide association study identified susceptible loci in vitiligo patients and found that these genes also have a strong association with diabetes mellitus, including *IFIH1, BACH2, BTNL2, IL2RA, SH2B3*, and *ZMIZ1* (118). Additionally, it has been revealed that elevated levels of the serum pro-inflammatory cytokines TNF-α, IL-1, and IL-6 play a role in the pathogenesis of vitiligo (119). The increase in these pro-inflammatory cytokines in serum and adipose tissue is also associated with insulin resistance and atherosclerosis. Furthermore, as mentioned above, melanocytes have been detected in adipose tissue. In vitiligo, lipid peroxidation causes a deterioration in reactive oxygen species, leading to a reduction in melanocytes in adipose tissue (108). Excessive reactive oxygen species contribute to adipogenesis by facilitating the proliferation and differentiation of pre-adipocytes and obesity (109), and decreased melanocyte numbers and melanogenesis impair their anti-inflammatory and antioxidative functions. These changes may predispose patients to develop MetS.

Recently, the association between vitiligo and MetS has attracted the attention of researchers, which further confirms that vitiligo not only affects the skin, but also that it has several systemic manifestations. MetS is an alarming health problem that increases the risk of type 2 diabetes mellitus and cardiovascular diseases, which can result in serious complications, such as myocardial infarction or stroke. Therefore, dermatologists should recognize the possibility of MetS in patients with vitiligo and refer patients to other specialists for further evaluation. Additionally, controlling risk factors, including obesity, high blood glucose, and high blood pressure, as well as maintaining a healthy lifestyle, may be beneficial for patients with vitiligo.

## 6. Psychological diseases

To the best of our knowledge, vitiligo greatly affects psychosocial well-being. Vitiligo patients experience a higher level of burden compared with healthy people, as reflected by quality-of-life (QoL) indicators. Morrison et al. (120) conducted a meta-analysis of 12 studies and demonstrated QoL impairment in patients with vitiligo, and subgroup analysis showed that those with darker skin or from Southern Asian cultures were more likely to suffer from reduced QoL. Additionally, psychosocial comorbidities were more prevalent in patients with vitiligo than in those with acne, AA, atopic dermatitis, and urticaria (121). One study reported that QoL impairments were even comparable to those observed in non-dermatologic diseases, such as chronic lung disease, arthritis, and cancer, according to SF-36 mental component scores (122).

Depression and anxiety are the most commonly reported psychosocial comorbidities. Wang et al. (123) showed that the prevalence of clinical depression was 8% and that of depressive symptoms increased to 33% among vitiligo patients. In addition, the patient group was 4.96 times more likely to suffer from depression symptoms compared with healthy people. Regarding anxiety, Kussainova et al. (124) suggested that the general incidence of anxiety in vitiligo patients was 35.8%, and the incidence was substantially higher among female patients.

Ezzedine et al. (121) also investigated the relationship between vitiligo and other psychological disorders. The most prevalent psychosocial comorbidities included feelings of stigmatization, sleep disturbance, adjustment disorders, avoidance or restriction behavior, and relationship difficulties, including sexual dysfunction. Moreover, several factors were associated with significantly higher psychological burden, including female sex, visible or genital lesions, age < 30 years (particularly adolescents), and extensive BSA involvement. Vitiligo affects not only the patient themself but also their caregivers. Parents of affected children were found to suffer from moderately reduced QoL and higher risks of depression and anxiety than parents of unaffected children (125). Intriguingly, a Korean multicenter, cross-sectional, prospective survey suggested that the willingness to pay was highest in vitiligo patients compared with that in subjects with other chronic skin conditions, including psoriasis, AD, AA, rosacea, chronic urticaria, and seborrheic dermatitis (126).

In summary, vitiligo can increase the psychological burden of patients and cause several psychological problems. Simultaneously, mental and psychological stress can further aggravate the progression of vitiligo, thus creating a positive feedback effect. Moreover, the degree of inflammation may be enhanced under emotional stress, which in turn contributes to the development of other comorbidities associated with vitiligo (109). Therefore, regular assessments of QoL and psychosocial state should be incorporated in routine clinical evaluation.

## 7. Conclusion

There is evidence in the literature that vitiligo is associated with several comorbid disorders (Figures 1, 2). However, more prospective studies and basic mechanism studies are needed to confirm these findings. These associations highlight the importance of a multidisciplinary approach in managing vitiligo patients. Dermatologists should consider these diseases associated with vitiligo to identify and screen potential co-morbidities in patients in a timely manner.

## Author contributions

ZH performed the literature search and drafted the first version of the manuscript. TW provided expert guidance and critically revised the work. All authors contributed to the article and approved the submitted version.

## Funding

This work was supported by National High Level Hospital Clinical Research Funding (2022-PUMCH-B-092), National High Level Hospital Clinical Research Funding (2022-PUMCH-A-164), Beijing Municipal Natural Science Foundation (Z210017), and Peking Union Medical College Hospital (ZC201911051).

## Conflict of interest

The authors declare that the research was conducted in the absence of any commercial or financial relationships that could be construed as a potential conflict of interest.

## Publisher’s note

All claims expressed in this article are solely those of the authors and do not necessarily represent those of their affiliated organizations, or those of the publisher, the editors and the reviewers. Any product that may be evaluated in this article, or claim that may be made by its manufacturer, is not guaranteed or endorsed by the publisher.
